# A Case Associated with Comorbidities Among Cerebral Infarction, Idiopathic Thrombocytopenic Purpura, and Triple X Syndrome

**DOI:** 10.4274/tjh.2013.0064

**Published:** 2014-06-10

**Authors:** Hanjun Kim, Sang Sun Hwang, Young Uh, Juwon Kim, Kap Jun Yoon, Ji-Yong Lee

**Affiliations:** 1 Yonsei University Wonju College of Medicine, Department of Laboratory Medicine, Wonju, Korea; 2 Yonsei University Wonju College of Medicine, Department of Neurology, Wonju, Korea

**Keywords:** Cerebral infarction, Idiopathic thrombocytopenic purpura, Triple X syndrome

## Abstract

A 46-year-old female presented to the emergency room due to the chief complaint of left-sided weakness. By imaging study, she was diagnosed with cerebral infarction. Thrombolytic and antiplatelet agents were not considered due to the “golden hour” for treatment having passed and a low platelet count. The peripheral blood smear, bone marrow biopsy, and aspirate findings were consistent with immune thrombocytopenic purpura. The chromosome analysis revealed the 47,XXX karyotype. To the best of our knowledge, this is the first case report associated with the comorbidities of cerebral infarction, idiopathic thrombocytopenic purpura, and triple X syndrome.

## Abstract (tr)

Kırk altı yaşındaki kadın hasta başlıca şikayeti sol tarafındaki zayıflık olarak acil servise başvurdu. Görüntüleme çalışmaları sonucu serebral enfarktüs tanısı kondu. Tedavi için değerli “altın saatler”in geçmiş olması ve düşük trombosit değerleri sebebiyle trombolitik tedavi ve antiagregan tedavi başlanmadı. Hastanın çevre kanı yayması, kemik iliği biyopsisi ve aspirasyonu immün trombositopenik purpura ile uyumluydu. Kromozom incelemesinde 47,XXX karyotip tayin edildi. Bilgilerimize göre, bu olgu serebral enfarkt, idyopatik trombositopenik purpura komorbiditelerinin birlikte bulunduğu ilk triple X sendromudur.

## INTRODUCTION

Triple X syndrome, also known as trisomy X (47,XXX karyotype), was first described in 1959 by Jacobs et al. [1]. It is relatively common as it occurs in about 1/1000 live female births. The cause of the disease is nondisjunction during the meiotic period and it is connected to advanced maternal age [2]. 

Idiopathic thrombocytopenic purpura (ITP) has a similar incidence of approximately 6/100.000 a year in the United States and United Kingdom. Pathophysiology of the disease is thought to be the destruction and impaired production of platelets by autoantibodies [3]. Recent studies indicate that genes located on the X chromosome play a major and unique role in autoimmunity [4]. The number of X chromosomes and the consequently altered X-linked gene dosage are critical for the maintenance or the loss of immune tolerance [5]. 

ITP is usually associated with bleeding tendency, but it also has thrombotic complications, which have been reported at rare intervals [3,6,7]. According to a recent large retrospective analysis, the incidence of thromboembolic events was 6.9% in ITP patients [3]. 

Recently, we experienced a case of cerebral infarction in a female ITP patient, and her karyotype revealed trisomy X. To the best of our knowledge, this is the first case presentation concerning comorbidities among triple disease entities: cerebral infarction, idiopathic thrombocytopenic purpura, and triple X syndrome. 

## CASE PRESENTATION

**Figure 1:**
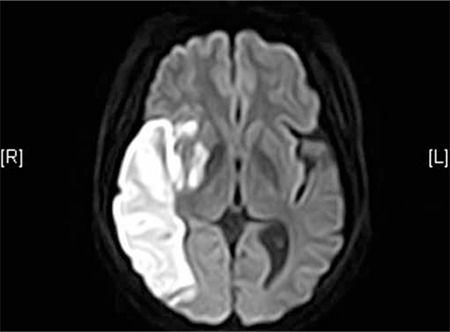
Large recent infarction involving right middle cerebral artery territory of frontoparietotemporal area and right striatocapsular area with mass effect and vascular enhancement.

**Figure 2:**
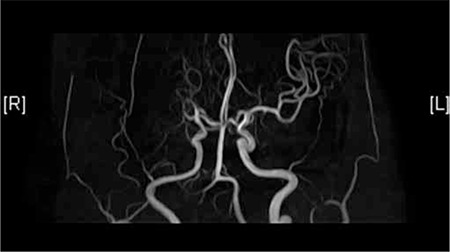
Right middle cerebral artery occlusion, steno-occlusive lesion involving right vertebral artery at v3-v4 segment.

A 46-year-old female was admitted to the emergency room due to the chief complaint of left-sided weakness that lasted for 10 h. She was a heavy lifelong drinker and smoker, but had no other medical history of note. On physical examination, all her vital signs were stable. She complained of dysarthria, facial weakness, headache, left-sided weakness, and visual field defect. By conducting a neurologic examination, right-sided gaze preponderance was seen, and visual field defect, facial weakness, and sensory change were detected on the left side. The motor function grades of the left-upper and left-lower extremities were also weakened to 2 and 4, respectively. The Babinski sign was positive on the left side. Contrast brain computed tomography and diffusion-weighted brain magnetic resonance imaging demonstrated recent infarction in the right middle cerebral artery territory (Figure 1). Contrast-enhanced magnetic resonance angiography was also done (Figure 2). Transesophageal echocardiography showed mitral regurgitation. Pelvic ultrasound showed no abnormal findings. Informed consent was obtained.

**Figure 3:**
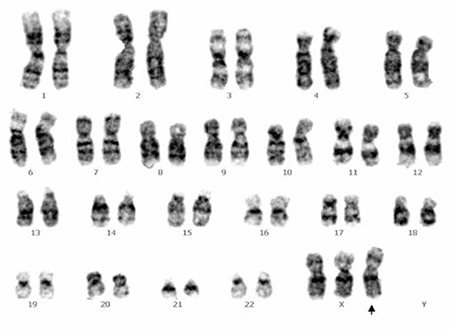
Chromosome analysis by G-banding technique revealed 47,XXX karyotype.

Since administration of thrombolytic agents was not considered due to the “golden hour” for treatment having passed by 3 h, she was treated with aspirin to prevent recurrence of cerebral infarction, and antiplatelet agents were withheld due to a low platelet count. The initial complete blood count was as follows: hemoglobin 11.7 g/L; WBC count 10.0x109/L with a differential count of 70% neutrophils, 23% lymphocytes, and 3.6% monocytes; and platelet count 20x109/L. To identify the cause of the low platelet count, a peripheral blood smear was done. It revealed normocytic anemia and thrombocytopenia. Bone marrow biopsy and aspiration findings were compatible with ITP, showing no evidence of malignancy but slightly increased megakaryocytes with some atypical forms. Antiplatelet antibody was positive. She was diagnosed with ITP and acute cerebral infarction. Chromosome analysis of the peripheral blood and bone marrow demonstrated a nonmosaic 47,XXX karyotype (Figure 3). Because of positive lupus anticoagulant, antiphospholipid syndrome (APS) was also considered, but her conditions did not meet the diagnostic criteria for APS because the lupus anticoagulant became negative upon follow-up laboratory assessment. Activity of factor VIII and antithrombin III, and protein C and protein S antigen levels and homocysteine level, were within the reference ranges. Protein factor VIII antibody was not detected. 

The aspirin therapy was discontinued when her platelet count recovered to above 50.000/µL, and intravenous dexamethasone (20 mg, twice daily) was started. About 1 month after admission, her general condition, including the neurologic symptoms, improved significantly and she was discharged from the hospital.

## DISCUSSION

Triple X patients generally experience normal pubertal development, but they can also suffer from mild developmental delays, behavioral problems, and learning disabilities [2]. In the present case, the patient and her 3 children did not experience mental or physical problems. It is supposed that most affected women are not identified but that cases are often diagnosed incidentally due to the low detection rate of triple X fetuses in prenatal ultrasounds, as well as the mild and variable phenotypic expression of the disease [2,8,9]. Concerning this karyotype, various functional and structural genitourinary abnormalities have been reported. They include bilateral polycystic kidney, hydronephrosis due to ureteral stricture, menstrual irregularity, and ovarian dysgenesis with urinary tract malformation in stillborn trisomy X fetuses [2,10,11]. Other abnormalities have also been mentioned: auditory defect, bilateral optic atrophy, brain anomaly, chorioretinitis, craniofacial dysmorphy, duodenal atresia, epileptic seizure, heart defect, jejunal atresia, and skeletal system anomalies [11]. 

Meanwhile, there are some reports on trisomy X associated with autoimmunity: 1 case with increased antinuclear antibody (ANA) and microsomal antithyroid antibody titers, 2 cases with systemic lupus erythematosus (SLE), 1 case with positive rheumatoid factor, and 1 ITP case with positive ANA and anticardiolipin antibody [2]. The higher prevalence of autoimmune diseases in women compared to men could be due to effects of ovarian hormones, pregnancy, and the presence of a second X chromosome [12]. One of the 2 X chromosomes in females undergoes inactivation during embryonic development. An inactivated X chromosome (XCI) may escape presentation of X-linked self-antigens in the thymus or in other peripheral sites that are involved in tolerance induction [13]. XCI may also be skewed during thymic development, resulting in predominant expression of only one set of X-chromosome-encoded self-antigens [12,14]. This may lead to inadequate thymic deletion of autoreactive T lymphocytes, which in turn leads to impaired self-antigen recognition and tolerance [13], thus triggering an autoimmune response in target cells. It is also possible that portions of the XCI can reactivate. According to recent studies, several X-chromosome-located microRNAs have important functions in immunity and thus possibly contribute to sex-specific immune responses [12,15]. Dai et al. [16] reported that 13 microRNAs had the same expression patterns in both SLE and ITP, which might generally be associated with autoimmune diseases. Additionally, 6 microRNAs that were only downregulated in ITP might be correlated with the organ-specific destruction of thrombocytes. Although there was no apparent causal connection between trisomy X and ITP, we hypothesize that as the number of X chromosomes increases, frequencies of escaping and/or skewing of XCI, and the expression level of microRNAs related to ITP, will increase. Further studies are necessary to confirm our hypothesis. 

An earlier study showed evidence that thrombotic events are related to antiphospholipid antibodies [7]. In this case, we could assume such a relationship due to positive lupus anticoagulant. Destructed platelets release humoral factors and platelet microparticles (PMPs) by an immunologic mechanism [7]. These PMPs induce activation of coagulation factors [17]. In other words, PMPs act as procoagulants and may protect against bleeding and promote thrombotic events in immunological thrombocytopenic patients [18]. Although it remains unclear whether these diseases had incidentally occurred or were predisposed by the additional X chromosome, we can hypothesize that the additional X chromosome may play an important role in autoimmunity. However, we could not clarify why the thrombus of the patient and ITP were associated with each other because hereditary thrombotic risk factors including the molecular studies of MTHFR, prothrombin, and factor V Leiden genes were not evaluated due to losing the patient to follow-up. 

Though therapy for ischemic stroke in ITP patients is still controversial, it would be better to manage patients according to the individualized pathophysiological mechanisms of the disease [6]. In a case similar to ours, successful results were made possible by improving platelet levels with steroids and intravenous immunoglobulin in the beginning, followed by treatment with antiplatelet agents [3]. 

As far as we know, this is the first case report associated with comorbidities among cerebral infarction, idiopathic thrombocytopenic purpura, and Triple X syndrome. 

## CONFLICT OF INTEREST STATEMENT

The authors of this paper have no conflicts of interest, including specific financial interests, relationships, and/or affiliations relevant to the subject matter or materials included.
